# The Role of C-Reactive Protein in Acute Myocardial Infarction: Unmasking Diagnostic, Prognostic, and Therapeutic Insights

**DOI:** 10.3390/jcm14134795

**Published:** 2025-07-07

**Authors:** Andreas Mitsis, Stefanos Sokratous, Georgia Karmioti, Michaela Kyriakou, Michail Drakomathioulakis, Michael M. Myrianthefs, Christos Eftychiou, Nikolaos P. E. Kadoglou, Stergios Tzikas, Nikolaos Fragakis, George Kassimis

**Affiliations:** 1 Cardiology Department, State Health Services Organization, Nicosia General Hospital, 215 Old Road Nicosia-Limassol, Nicosia 2029, Cyprus; stefanossokratous94@gmail.com (S.S.); georgiakarm@outlook.com (G.K.); michaelakyriakou95@yahoo.com (M.K.); bageragr@gmail.com (M.D.); mmyrianthefs@gmail.com (M.M.M.); chr6eft@gmail.com (C.E.); 2 Medical School, University of Cyprus, Nicosia 2115, Cyprus; nikoskad@yahoo.com; 3 Third Department of Cardiology, Aristotle University of Thessaloniki, 54626 Thessaloniki, Greece; 4 Second Department of Cardiology, Aristotle University of Thessaloniki, 54642 Thessaloniki, Greece; fragakis.nikos@gmail.com (N.F.); gksup@yahoo.gr (G.K.)

**Keywords:** C-reactive protein, acute myocardial infarction, inflammation, biomarkers, cardiovascular risk, prognosis, diagnosis, immune response

## Abstract

C-reactive protein (CRP) has emerged as a valuable biomarker in acute myocardial infarction (AMI), offering multiple insights into diagnosis, prognosis, and therapeutic strategies. In the diagnostic domain, elevated CRP levels serve as an early indicator of AMI, aiding in prompt identification and initiation of treatment. Prognostically, CRP is a strong predictor of adverse outcomes post-AMI, correlating with increased mortality and cardiovascular events. Beyond its diagnostic and prognostic roles, CRP also exposes therapeutic avenues in AMI management. Targeting CRP through pharmacological interventions has shown promise in reducing inflammatory responses, thereby mitigating myocardial damage and improving clinical outcomes. However, CRP’s low specificity, influenced by elevation in non-cardiac conditions, remains a clinical limitation that warrants consideration. This review comprehensively examines the evolving role of CRP in AMI, exploring its diagnostic accuracy, prognostic significance, and potential as a therapeutic target. The understanding of the complex role of CRP in AMI provides clinicians with valuable tools for risk stratification, treatment optimization, and personalized patient care in the acute setting.

## 1. Introduction

C-reactive protein (CRP) is a critical acute-phase protein produced by the liver in response to inflammation [1]. It plays a significant role in the body’s immune response, serving as a sensitive marker of systemic inflammation and tissue damage [2]. Elevated levels of CRP have been implicated in various inflammatory and cardiovascular diseases, particularly acute myocardial infarction (AMI) [3]. The role of CRP in cardiovascular pathology has been extensively studied, revealing its potential as a biomarker for diagnosis, prognosis, and therapeutic targeting [4]. In the context of AMI, inflammation is a key pathological process that contributes to myocardial injury, adverse left ventricular (LV) remodeling, and heart failure [5]. Understanding the dynamics of CRP levels post-AMI and their correlation with clinical outcomes is crucial for improving patient management and developing targeted therapeutic strategies.

**Figure 1:**
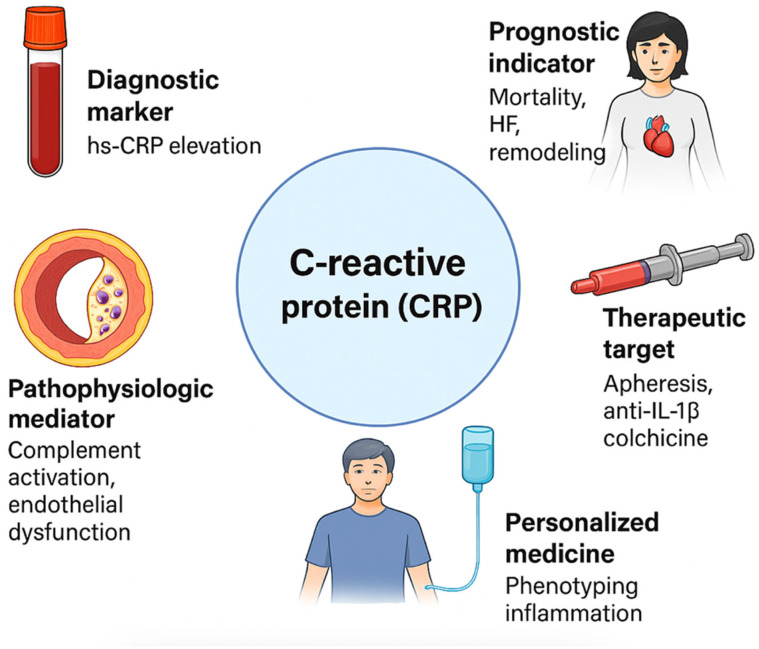
Multifaceted roles of C-reactive protein (CRP) in acute myocardial infarction. CRP: C-reactive protein; HF: heart failure; hs-CRP: high-sensitivity C-reactive protein; IL-1β: interleukin-1 beta.

The purpose of this manuscript is to provide a comprehensive review of the diagnostic, prognostic, and therapeutic roles of CRP in AMI (Figure 1). By examining the latest research and clinical studies, this manuscript aims to expose the significance of CRP as a biomarker in AMI, discuss its potential as a therapeutic target, and highlight the challenges and controversies surrounding its clinical application. This review also explores the implications of CRP measurement for risk stratification and clinical decision-making in AMI, offering insights into future research directions and emerging therapeutic approaches that could enhance patient outcomes.

## 2. Search Strategy

A comprehensive narrative literature review was conducted to synthesize the current knowledge on the role of C-reactive protein (CRP) in acute myocardial infarction (AMI). The search was performed across major electronic databases, including PubMed, Scopus, and Web of Science, covering articles published up to March 2025. Relevant keywords and combinations such as “C-reactive protein,” “CRP,” “acute myocardial infarction,” “acute coronary syndromes,” “inflammation,” and “prognosis” were utilized. Both original research articles and systematic reviews were considered, with emphasis placed on clinical studies, translational research, and experimental data exploring the pathophysiological mechanisms, diagnostic utility, and prognostic significance of CRP in AMI. Preference was given to high-impact, peer-reviewed publications in English. Studies focusing on the relationship between CRP levels and infarct size, left ventricular remodeling, recurrent ischemic events, and overall clinical outcomes were prioritized. Articles involving pediatric populations, case reports, and non-cardiac applications of CRP were excluded. Manual screening of reference lists from key articles was also undertaken to identify additional pertinent studies. The findings from the selected literature were critically analyzed and synthesized to provide an updated and integrative overview of CRP’s clinical and biological relevance in acute myocardial infarction.

## 3. The Inflammatory Response After an AMI

The inflammatory response following an AMI is a double-edged sword, playing a vital role in tissue repair while also posing risks if dysregulated [6]. This complex process involves a finely tuned interplay between various immune cells and inflammatory molecules, with macrophages being central to this response [7].

Upon the occurrence of AMI, the immediate response includes the infiltration of neutrophils into the infarcted myocardium [8]. Neutrophils are among the first responders, releasing reactive oxygen species (ROS) and proteolytic enzymes that help in clearing necrotic tissue [9,10]. However, their presence is short-lived as they can also exacerbate tissue damage if their activity is prolonged [11]. Macrophages are the key players in the subsequent phases of the inflammatory response [12]. These cells can exist in different phenotypic states, broadly categorized into pro-inflammatory (M1) and anti-inflammatory (M2) macrophages [13]. M1 macrophages dominate the early phase post-AMI, secreting pro-inflammatory cytokines such as tumor necrosis factor—α (TNF-α), interleukin (IL) -1β, and IL-6, which are crucial for debris clearance and initiating tissue repair processes [14]. As the healing progresses, there is a phenotypic switch from M1 to M2 macrophages, which release anti-inflammatory cytokines like IL-10 and transforming growth factor beta (TGF-β), promoting the resolution of inflammation and tissue remodeling [15]. Other immune cells, including lymphocytes and dendritic cells, also contribute to the inflammatory milieu. Regulatory T cells (Tregs) [16] and myeloid-derived suppressor cells (MDSCs) [17] play roles in dampening the inflammatory response and aiding in the resolution phase [18].

The balance between pro-inflammatory and anti-inflammatory molecules is crucial for an optimal healing process [19]. Pro-inflammatory cytokines such as TNF-α, IL-1β, and IL-6 initiate and sustain the inflammatory response, facilitating the removal of dead cells and pathogens [20,21]. Chemokines like MCP-1 (monocyte chemoattractant protein-1) attract monocytes to the site of injury, where they differentiate into macrophages [22]. On the other hand, anti-inflammatory molecules such as IL-10 [23] and TGF-β [24] are essential for controlling and resolving the inflammatory response. These molecules inhibit the activity of pro-inflammatory cytokines, promote the clearance of apoptotic cells, and encourage tissue repair and fibrosis [25].

Maintaining a balance between pro-inflammatory and anti-inflammatory responses is critical. An excessive pro-inflammatory response can lead to chronic inflammation, extensive tissue damage, and adverse remodeling of the heart, potentially resulting in heart failure. For instance, high levels of TNF-α and IL-6 are associated with worse outcomes and increased myocardial injury [26,27]. Conversely, an inadequate inflammatory response can impair the clearance of necrotic tissue and delay healing. Anti-inflammatory molecules like IL-10 and TGF-β play protective roles by limiting tissue damage, reducing fibrosis, and promoting healing [24,28].

Excessive pro-inflammatory action can lead to adverse outcomes including elevated myocardial injury and infarct size, an increased risk of cardiac rupture, adverse ventricular remodeling, and finally the development of heart failure [29]. Conversely, the preventive role of anti-inflammatory molecules includes the limiting the extent of inflammation and tissue damage, the promotion of the resolution of inflammation, the facilitation of tissue repair and regeneration, and finally, reducing the risk of adverse remodeling and heart failure [30].

Oxidative stress in AMI is driven by an excessive production of ROS, which damage cellular components and activate inflammatory pathways [31]. ROS stimulate the release of pro-inflammatory cytokines and trigger the activation of transcription factors like NF-kB, promoting the production of CRP in the liver. This elevated CRP, in turn, exacerbates vascular inflammation by amplifying the recruitment of immune cells and enhancing endothelial dysfunction, contributing to further myocardial damage [32]. In addition, the NOD-like receptor protein 3 (NLRP3) inflammasome plays a critical role in CRP-mediated inflammation during AMI by activating pro-inflammatory cytokines, particularly IL-1β and IL-18 [33]. Upon activation by stress signals such as oxidative stress and mitochondrial damage, NLRP3 triggers the cleavage of pro-caspase-1 into its active form, which in turn processes pro-IL-1β and pro-IL-18 into their active inflammatory forms. This cascade leads to an enhanced inflammatory response, promoting leukocyte infiltration and contributing to myocardial injury and tissue damage in AMI [34].

Several studies have demonstrated that inflammation in the initial days following AMI can predict subsequent functional decline and influence therapeutic decisions [5]. Thackeray et al. highlighted that molecular imaging using cardiac positron emission tomography (PET)/computed tomography (CT) scan synchronizes diagnosis and therapy by visualizing inflammation post-MI and predicting cardiac remodeling [35]. The post-MI inflammatory response, if accentuated, prolonged, or expanded, can lead to adverse LV remodeling, myocardial dysfunction, and heart failure. Saxena et al. discussed the fact that persistent inflammation post-MI exacerbates myocardial injury and promotes adverse cardiac remodeling [36]. Moreover, specific cellular and molecular processes, such as the involvement of dendritic cells and Toll-like receptor 4 (TLR4), have been identified as key regulators of post-infarction inflammation [37,38]. MicroRNAs and signaling pathways, including the phosphoinositide-3-kinase (PI3K)/serine-threonine kinase (Akt)/nuclear factor kappa B (NF-κB) pathway, have also been implicated in reducing vascular inflammation and apoptosis in the context of MI. Zhao et al. demonstrated that microRNA-146b modulates these pathways to mitigate inflammatory responses and improve myocardial cell survival post-MI [39]. This complex interplay between various inflammatory mediators (Table 1) underscores the significance of targeting inflammation in managing AMI and improving patient outcomes [40].

## 4. The Diagnostic Role of CRP in Cardiovascular Diseases

High-sensitive CRP (hs-CRP) assays were developed to detect subtle fluctuations in plasma CRP levels, and elevated hs-CRP concentrations have been linked to an increased risk of cardiovascular events. Serum hs-CRP could be a useful biomarker for indicating coronary artery disease (CAD) severity and could aid in risk stratification [41]. Typically, hs-CRP levels of ≥2 mg/L are considered a threshold for heightened cardiovascular risk [42]. Incorporating hs-CRP into cardiovascular risk prediction models, such as the Reynolds Risk Score, has shown promising results [43]. Studies have demonstrated that both very high and very low CRP values can be clinically useful for risk stratification [44]. Among them, the Justification for the Use of Statins in Prevention: an Intervention Trial Evaluating Rosuvastatin (JUPITER) trial, indicated that elevated hs-CRP levels were a significant factor contributing to higher cardiovascular event rates, independent of other risk factors [45].

CRP is an essential biomarker for inflammation, which can indicate the severity of AMI. It has been suggested that CRP directly contributes to the pathogenesis of inflammation and atherosclerosis [46]. CRP has high sensitivity and specificity for detecting inflammation. Therefore, CRP has been used as a marker of inflammation in various clinical settings of an acute coronary syndrome (ACS) [47]. In the settings of an AMI, CRP levels rise in response to myocardial necrosis, reflecting the extent of inflammation and tissue damage. CRP usually peaks 24–72 h after symptom onset and, often, elevated CRP levels are indicative of the acute inflammatory response triggered by MI [48,49]. This rise in CRP is mediated by cytokines such as IL-6, which are released from the necrotic myocardium and activate the liver to produce CRP [50]. High CRP levels correlate with the severity of myocardial injury and are associated with worse clinical outcomes, including an increased risk of adverse cardiac events and mortality [51,52]. Furthermore, increased serum levels of hs-CRP appeared to be strong predictors of high SXScore in ACS patients [53]. Monitoring CRP levels in patients with AMI provides valuable prognostic information, helping to identify those at higher risk of complications and guiding therapeutic decisions to mitigate further damage [54]. Interestingly, many studies have shown a direct correlation between salivary CRP levels and AMI, suggesting its potential in cardiovascular risk assessment [55,56,57].

In conclusion, CRP could be used to assess cardiovascular risk and detect patients at risk of MI [58]. However, currently, hs-CRP is not recommended for decision-making purposes, and data on its distribution remain limited. A significant drawback of this marker is its lack of specificity [59,60]. There is no standardized cutoff to distinguish between levels associated with infection versus atherosclerosis or ACS, although the literature suggests values more than ten times higher than the commonly referenced threshold of 2 mg/L [61].

## 5. Prognostic Role of CRP in AMI

Higher CRP levels post-AMI are significantly associated with adverse outcomes, including early and late clinical events, cardiogenic shock, and heart failure [62,63,64]. Studies have demonstrated that human CRP production increases significantly after AMI and peak post-infarction plasma levels of CRP are strongly associated with early and late clinical outcomes [65] and even 30-day mortality [66]. CRP levels correlate with the severity of CAD and can predict complications such as infarct expansion and cardiac rupture [67]. Furthermore, associations of serum hs-CRP with coronary vessel stenosis and heart failure in AMI patients have been reported, indicating its potential relevance in assessing cardiovascular complications [68]. Interestingly, a positive correlation has been found between CRP and troponin values and ventricular arrhythmias in AMI, underscoring the potential of CRP as a prognostic indicator in malignant arrythmias and sudden cardiac death after AMI [69,70].

CRP appears to correlate not only with short term, but also with long term prognoses post-AMI. Long-term studies indicate that CRP is a reliable predictor of heart failure and left ventricular remodeling post-AMI, underlining its prognostic value [71]. Interestingly, very early blood CRP levels in patients with AMI have been shown to predict functional capacity, LV function, extent of CAD, early and short-term complications, and 1-year mortality [72]. Similarly, CRP has been linked to LV remodeling and function after AMI, suggesting its prognostic value in predicting late outcomes [73]. Of note, a high hs-CRP level measured after the first AMI predicts myocardial dysfunction and heart failure and it is suggested that hs-CRP plays an important role in the development of heart failure after myocardial infarction [74]. Moreover, CRP has been shown to be associated with the presence and severity of mitral regurgitation and diastolic dysfunction in the initial phase of AMI [75]. These studies underscore the potential utility of CRP as a valuable biomarker for risk stratification and prognostication, and for understanding the underlying mechanisms of myocardial injury in the context of AMI (Table 2).

## 6. CRP as a Treatment Target in AMI

In recent years, targeting inflammation through CRP modulation has gained attention as a potential strategy to reduce myocardial injury and improve outcomes in patients with AMI [76]. Therapeutic interventions aimed at modulating CRP or reducing its inflammatory effects hold promise. One such approach is CRP apheresis, a method that directly removes CRP from circulation, potentially limiting its detrimental effects on the myocardium during AMI [77]. Early experimental studies in pigs have shown that CRP apheresis may reduce myocardial injury, preserve cardiac function, and improve long-term outcomes [78,79,80]. While further clinical trials are needed to establish its efficacy, this technique represents a novel and direct method of CRP reduction [81]. The recently published CRP apheresis in Acute Myocardial Infarction study (CAM)-1 study showed that lowering CRP levels by apheresis resulted in the loss of correlation of CRP concentrations with myocardial infarct sizes as well as LV function [82].

In addition to apheresis, anti-inflammatory pharmacological agents have been explored to mitigate CRP-related myocardial damage. Colchicine, a well-known anti-inflammatory agent, has shown promise in targeting CRP-driven inflammation and reducing major advert cardiac events (MACE)s post-AMI [83]. The COLCOT (Colchicine Cardiovascular Outcomes Trial) [84] and LoDoCo2 (Low-Dose Colchicine) [85] studies have compellingly highlighted the significant impact of low-grade inflammation on the prognosis of AMI patients in relation to future cardiovascular events. Colchicine therapy has demonstrated a reduction in mortality by effectively lowering inflammation, particularly by reducing CRP levels [86].

Similarly, the results of the CANTOS (Canakinumab Anti-inflammatory Thrombosis Outcomes Study) trial provided significant evidence for the role of inflammation in post-AMI outcomes [87]. In this large-scale clinical trial, canakinumab, an IL-1ß inhibitor, was shown to reduce recurrent cardiovascular events in patients with a history of AMI by specifically targeting inflammation, without affecting lipid levels. One of the key findings was the substantial reduction in CRP levels, demonstrating that targeting inflammation independently of cholesterol management could have a profound effect on reducing cardiovascular risk. The reduction in CRP correlated with a decrease in MACEs, underscoring the role of inflammation as a critical therapeutic target in post-AMI management [88]. However, the study also highlighted the potential for increased infection risk due to the suppression of the immune system, which is a consideration for long-term therapy.

Other agents, such as tacrolimus and dexamethasone, have demonstrated potent anti-inflammatory properties, making them potential adjunctive therapies in managing inflammation in AMI [89]. As a calcineurin inhibitor, tacrolimus primarily suppresses T-cell activation by inhibiting the transcription of inflammatory cytokines such as IL-2 [90]. By reducing the activation of T-cells and dampening the inflammatory cascade, tacrolimus may help prevent further myocardial damage post-AMI [91]. However, its use is not without challenges, as it carries a risk of nephrotoxicity, metabolic complications, and an increased susceptibility to infections due to its broad immunosuppressive effects.

Dexamethasone exerts anti-inflammatory effects by inhibiting multiple inflammatory pathways, including the suppression of pro-inflammatory cytokines (such as IL-1 and IL-6) and the modulation of NF-kB signaling [92]. In the context of AMI, dexamethasone has the potential to reduce inflammation-related myocardial injury and lower CRP levels [93]. Its rapid action in controlling inflammation makes it an appealing option, particularly in acute settings [94]. However, prolonged use can lead to significant systemic side effects, such as hyperglycemia, hypertension, and immunosuppression, which must be carefully managed in patients post-AMI [95].

Stem cell-derived exosomes have emerged as a promising therapeutic approach for reducing inflammation and potentially lowering CRP levels in AMI. These exosomes are small extracellular vesicles secreted by stem cells that carry bioactive molecules, including proteins, lipids, and microRNAs, which can modulate cellular responses and promote tissue repair [96]. Research has shown that stem cell-derived exosomes can exert potent anti-inflammatory effects by regulating immune cell activity and dampening pro-inflammatory signaling pathways [97]. In the context of AMI, exosomes derived from mesenchymal stem cells have demonstrated the ability to reduce myocardial injury by inhibiting the release of inflammatory cytokines and attenuating the systemic inflammatory response [98], which includes lowering CRP levels. These exosomes can also promote cardiomyocyte survival and enhance cardiac repair by activating regenerative pathways and limiting the extent of ischemic damage [99,100]. While still in the early stages of clinical development, stem cell-derived exosome therapy represents a novel and exciting avenue for targeting CRP and inflammation, potentially improving outcomes for AMI patients by reducing the inflammatory burden and fostering myocardial healing.

Beyond general CRP reduction, attention has turned toward specific inflammatory pathways that drive the pathological effects of elevated CRP in AMI. Agents like α-lipoic acid [101,102], berberine [103], and astragalin [104] are being investigated for their anti-inflammatory properties, particularly in their ability to modulate oxidative stress and cytokine release. These compounds have shown potential in reducing myocardial injury by dampening the inflammatory response associated with AMI.

One key pathway involved in CRP-mediated inflammation is the NLRP3 inflammasome, which plays a central role in the activation of inflammatory cytokines such as IL-1β [105]. Modulation of the NLRP3 inflammasome has emerged as a potential therapeutic target, as inhibition of this pathway could suppress the inflammatory cascade and reduce myocardial damage [106,107]. Pharmacological agents that inhibit the NLRP3 inflammasome are currently under investigation, and early results suggest they may have a role in improving outcomes post-AMI [108].

As CRP continues to be explored as a treatment target in AMI, ongoing research is critical to identify the most effective strategies for its modulation (Table 3). Future studies should aim to refine CRP apheresis techniques and investigate combination therapies that integrate anti-inflammatory agents with traditional AMI management strategies. Personalized treatment approaches that consider CRP levels and individual inflammatory profiles may provide a more tailored and effective intervention for patients at risk of severe inflammatory complications post-AMI.

## 7. Challenges and Controversies

While CRP has been extensively studied as a biomarker in the context of AMI, its use remains fraught with challenges and controversies. One of the primary concerns is CRP’s low specificity [109]. As a general marker of inflammation, CRP levels can be elevated in various non-cardiovascular conditions, which act as confounders in the interpretation of CRP levels. These conditions include infections and sepsis [110], autoimmune diseases [111], chronic kidney disease [112], malignancies [113], obesity and metabolic syndrome [114], and trauma and surgery [115]. These conditions, among others, complicate the interpretation of CRP as a cardiovascular marker, as elevated levels may not specifically indicate myocardial inflammation. Relying solely on CRP to diagnose AMI can lead to false positives and diagnostic ambiguity. This nonspecific rise in CRP makes it difficult to ascertain whether elevated levels are solely indicative of cardiovascular inflammation in AMI patients or whether they reflect an unrelated inflammatory process. Consequently, reliance on CRP as a standalone diagnostic marker for AMI can lead to false positives and diagnostic ambiguity. To overcome the issue of non-specificity, combining CRP with biomarkers like IL-6 or other inflammatory biomarkers may increase diagnostic precision.

Another challenge lies in the timing of CRP elevation. CRP levels tend to peak approximately 24 to 72 h after the onset of symptoms in AMI [48], limiting its utility as an early diagnostic tool. In contrast, biomarkers such as troponins rise more rapidly following myocardial injury, making them more sensitive for the early detection of AMI [116]. CRP’s delayed elevation has raised questions about its role as a diagnostic marker in acute settings, where an early and accurate detection of myocardial infarction is crucial.

In terms of prognosis, while elevated CRP levels have been correlated with worse cardiovascular outcomes and recurrent events, the precise role of CRP in risk stratification remains controversial. Some studies suggest that CRP is an independent predictor of adverse outcomes, while others indicate that its prognostic value diminishes when adjusted for other well-established risk factors, such as cholesterol levels, blood pressure, and smoking status [117]. This has led to debates over the incremental benefit of measuring CRP in AMI patients, particularly when traditional risk markers are already being assessed.

Additionally, there is still uncertainty regarding CRP as a therapeutic target. While reducing CRP levels may reflect a reduction in inflammation, it is unclear whether interventions specifically aimed at lowering CRP confer any direct clinical benefit [118]. Trials targeting inflammation, such as those using IL-1β inhibitors, have demonstrated promise, but whether these effects are mediated by CRP reduction or broader anti-inflammatory mechanisms remains debated. Good patient selection, including those who experience extensive MIs, and consequently a significant inflammatory burden, may gain greater benefit from early modulating CRP interventions [119].

In summary, although CRP has potential as a marker in AMI, its low specificity, delayed elevation, and questionable prognostic utility limit its application in clinical practice. Further research is needed to clarify its role, particularly as a therapeutic target, and to determine whether it provides additional value over established cardiovascular risk markers.

## 8. Emerging Biomarkers in Combination with CRP

While CRP has been established as a marker of inflammation and cardiovascular risk, it lacks specificity and may not fully capture the complexity of the inflammatory response in AMI. To improve diagnostic accuracy and prognostic prediction, researchers are exploring the use of emerging biomarkers in combination with CRP [120]. For example, IL-6 has been shown to correlate with both the extent of myocardial damage and the severity of inflammation in AMI [121]. Combining IL-6 with CRP could provide a more comprehensive assessment of the inflammatory burden and improve risk stratification [122].

Another promising biomarker is procalcitonin, which is traditionally used to detect bacterial infections but has shown potential in distinguishing between low-grade chronic inflammation and acute inflammatory states, such as those seen in AMI [123,124,125]. Additionally, microRNAs, small non-coding RNA molecules, are gaining attention for their ability to regulate gene expression and reflect the underlying molecular mechanisms of inflammation and atherosclerosis [126]. Studies suggest that certain microRNAs, when used in conjunction with CRP, may help refine the diagnosis of AMI and offer insights into plaque stability and rupture [127]. The combination of CRP with these and other emerging biomarkers could enhance the specificity and predictive power of inflammatory markers in cardiovascular disease [128].

## 9. Personalized Medicine and CRP: Tailoring Treatment Strategies

With advancements in personalized medicine, CRP is emerging as a potential tool for tailoring treatment strategies in patients with AMI [129]. By integrating CRP levels into the risk stratification process, clinicians can better identify high-risk individuals who may benefit from more aggressive anti-inflammatory and cardiovascular therapies [130]. For instance, patients with persistently elevated CRP levels after AMI may require closer monitoring and more intensive interventions, such as the use of anti-inflammatory agents (e.g., colchicine [84] or IL-1β inhibitors [88]) or statins with a dual lipid-lowering and anti-inflammatory effect [131].

Personalized medicine in AMI could also involve targeting specific inflammatory pathways identified through CRP and other biomarkers [132]. The emergence of a distributive profile should be considered an early surrogate of systemic inflammatory response syndrome (SIRS) progression and impending multiorgan failure. Rapid recognition may prompt timely, targeted anti-inflammatory measures—cytokine adsorption, selective immunomodulation, metabolic support—and potentially prevent escalation to advanced SCAI stages D or E, where in-hospital mortality can exceed 90% [133]. For example, patients with elevated CRP and concomitant markers of oxidative stress or endothelial dysfunction may benefit from antioxidant therapies [134], while those with elevated CRP and NLRP3 inflammasome activation could be candidates for therapies targeting this specific pathway [108,135]. Pre-clinical work with dapansutrile (OLT-1177), a selective NLRP3 inhibitor, demonstrated marked infarct-size reduction and the preservation of systolic function when given within 60 min of reperfusion [136]. Additionally, genetic variations influencing CRP production or response to anti-inflammatory treatments may guide therapeutic decisions [137]. Single-nucleotide polymorphisms (SNPs) within the CRP locus influence baseline hs-CRP concentrations and modulate cardiovascular risk [138]. Further research is needed to explore genetic polymorphisms affecting CRP expression, the integration of CRP into multi-biomarker algorithms, and the role of AI in predicting outcomes based on inflammatory profiles. Recent interpretable machine-learning models that integrate hs-CRP with troponin, NT-proBNP, metabolomics, and clinical variables have achieved c-statistics ≥0.80 for predicting 30-day and long-term mortality after AMI [139].

Ultimately, incorporating CRP into a personalized treatment framework holds promise for improving outcomes by addressing the individual inflammatory profiles of AMI patients, thereby moving beyond the “one-size-fits-all” approach.

## 10. Conclusions

CRP has emerged as a multifaceted biomarker in the context of AMI, offering diagnostic, prognostic, and therapeutic insights. Elevated CRP levels serve as an early marker of inflammation and are closely associated with the extent of myocardial injury. Furthermore, CRP levels correlate with adverse outcomes, including left ventricular dysfunction, heart failure, and mortality, making it a valuable prognostic tool. In the therapeutic landscape, interventions targeting inflammation and CRP, such as CRP apheresis, colchicine, and IL-1β inhibitors, show promise in reducing cardiovascular events and improving outcomes in AMI patients. However, challenges remain, including CRP’s low specificity and timing of elevation, which limit its clinical application. Future research is needed to refine therapeutic strategies targeting CRP, optimize patient selection, and fully harness CRP’s potential as a biomarker and treatment target in AMI management.

## Figures and Tables

**Table 1:** Key molecular pathways involved in CRP-mediated inflammation in AMI.

Molecular Pathway	Key Components	Role in CRP-Mediated Inflammation
IL-1β Pathway	IL-1β, IL-6, NF-kB	Amplifies CRP expression and drives acute inflammatory responses post-AMI
NLRP3 Inflammasome	NLRP3, Caspase-1, IL-18	Activates inflammatory cytokines and contributes to myocardial damage
Oxidative Stress	ROS, NADPH Oxidase, NOX2	Promotes CRP production and enhances vascular inflammation
Toll-Like Receptors	TLR2, TLR4	Activates immune cells, increases CRP levels, and worsens myocardial injury
Monocyte Recruitment	MCP-1, CCR2	Recruits monocytes to inflamed areas, promoting macrophage activity and CRP release

CCR2: C-C Chemokine Receptor Type 2; IL-1β: Interleukin 1 Beta; MCP-1: Monocyte Chemoattractant Protein-1; NF-kB: Nuclear Factor Kappa-light-chain-enhancer of activated B cells; NLRP3: Nucleotide-binding oligomerization domain-like receptor protein 3; NOX2: NADPH Oxidase 2; ROS: Reactive Oxygen Species; TLR: Toll-Like Receptor.

**Table 2:** Studies correlating elevated C-reactive protein (CRP) levels after acute myocardial infarction (AMI).

Author	Study Design	Country	Population	Associated Outcomes	Study Findings
Griselli et al. (1999) [65]	Experimental study	UK	Animal model and human samples	Tissue damage	CRP and complement were found to be important mediators of tissue damage in AMI, suggesting that elevated CRP correlates with increased tissue damage.
Morishima et al. (2002) [73]	Prospective observational	Japan	250 AMI patients treated with coronary angioplasty	Left ventricular remodeling	High CRP levels (>2.0 mg/dL) were associated with poor left ventricular remodeling and decreased function following AMI (*p* < 0.05).
Bursi et al. (2007) [64]	Community-based cohort	USA	546 AMI patients	Heart failure post-AMI	Elevated CRP levels (>3 mg/L) were associated with an increased risk of heart failure in AMI patients (HR: 2.8; 95% CI: 1.9–4.3; *p* < 0.001).
Akkus et al. (2009) [67]	Prospective observational	Turkey	100 AMI patients	Cardiogenic shock, heart failure	High admission CRP levels (>10 mg/L) were associated with increased risk of cardiogenic shock (*p* = 0.001) and heart failure (*p* = 0.003) in AMI patients.
Ribeiro et al. (2014) [66]	Prospective cohort	Brazil	208 AMI patients (STEMI)	Cardiovascular events post-AMI	Hs-CRP (>3 mg/L) was a significant predictor of adverse cardiovascular events after STEMI (HR: 2.51; 95% CI: 1.33–4.74; *p* = 0.005).
Sharif et al. (2015) [72]	Prospective observational	Israel	150 AMI patients	Early and late prognosis	Elevated CRP levels (>2.5 mg/dL) immediately after AMI were predictive of adverse outcomes at 30 days and 1 year (*p* < 0.01 for all time points).
Kobayashi et al. (2018) [70]	Prospective observational	Japan	274 AMI patients	Electrical instability, in-hospital electrical storm	CRP >5 mg/L was associated with increased risk of electrical storm post-AMI (OR: 3.1; *p* < 0.01).
Al Aseri et al. (2019) [74]	Prospective observational	Saudi Arabia	200 first MI patients	Progression to heart failure	hs-CRP >3 mg/L significantly increased risk of heart failure post-first MI (HR: 2.27; 95% CI: 1.62–3.19; *p* < 0.001).
Świątkiewicz et al. (2021) [71]	Longitudinal observational	Poland	1000 post-AMI patients	Post-infarct heart failure	Discharge CRP > 3 mg/L predicted a >50% increased risk of heart failure at 5-year follow-up (*p* < 0.001).
Zhu et al. (2023) [68]	Retrospective cohort	China	300 AMI patients	Coronary vessel stenosis, heart failure	hs-CRP >2 mg/L was significantly associated with severe coronary artery stenosis (*p* = 0.003) and higher incidence of heart failure (*p* = 0.004).

AMI: Acute myocardial infarction; CRP: C-reactive protein; Hs: High-sensitive, STEMI: ST-elevation myocardial infarction; UK: United Kingdom; USA: United States of America.

**Table 3:** Therapeutic methods targeting inflammation in acute myocardial infarction (AMI).

Method	Mechanism of Action	Advantages	Limitations
CRP Apheresis	Direct removal of CRP from the bloodstream via apheresis	Rapid reduction of CRP levels, potential to limit myocardial damage	Limited clinical trials, cost, and invasive procedure
Colchicine	Anti-inflammatory agent that suppresses neutrophil activation	Reduces inflammation and CRP levels, oral administration, low cost	Gastrointestinal side effects, long-term safety in AMI patients unclear
IL-1ß Blockade	Inhibits the IL-1ß pathway, reducing systemic inflammation	Proven reduction in major adverse cardiovascular events (CANTOS)	High cost, increased risk of infections
Stem Cell-Derived Exosomes	Modulates immune response and promotes tissue repair	Non-invasive, regenerative potential, reduces systemic inflammation	Early-stage clinical development, unclear long-term outcomes
Tacrolimus	Suppresses immune activation and inflammatory pathways	Potent immunosuppressive and anti-inflammatory effects	Risk of nephrotoxicity, increased susceptibility to infections
Dexamethasone	Corticosteroid that inhibits multiple inflammatory pathways	Strong anti-inflammatory effects, reduces CRP levels	Immunosuppression, potential for hyperglycemia and other systemic side effects
α-Lipoic Acid	Antioxidant that reduces oxidative stress and inflammation	Low cost, oral administration	Limited evidence in AMI, may require combination with other therapies
Berberine	Reduces inflammation and modulates lipid metabolism	Anti-inflammatory and cardioprotective properties	Limited data in AMI-specific applications, gastrointestinal side effects
Astragalin	Anti-inflammatory agent with antioxidant properties	Natural compound with potential cardioprotective effects	Limited clinical data, unclear dosage and efficacy in humans
NLRP3 Inflammasome Modulation	Inhibits the NLRP3 inflammasome to prevent cytokine release	Targets a specific inflammatory pathway, potential for long-term benefits	Experimental stage, risk of off-target effects

AMI: Acute myocardial infarction; CANTOS: Canakinumab Anti-inflammatory Thrombosis Outcome Study; CRP: C-reactive protein; IL-1ß: Interleukin 1ß; NLRP3: NOD-, LRR- and pyrin domain-containing protein 3.

## Data Availability

No new data were created or analyzed in this study.

## References

[1] Kushner I. (2023). C-Reactive Protein—My Perspective on Its First Half Century, 1930-1982. Front. Immunol..

[2] Bhattacharya S., Munshi C. (2023). Biological Significance of C-Reactive Protein, the Ancient Acute Phase Functionary. Front. Immunol..

[3] Wilson A.M., Ryan M.C., Boyle A.J. (2006). The Novel Role of C-Reactive Protein in Cardiovascular Disease: Risk Marker or Pathogen. Int. J. Cardiol..

[4] de Ferranti S.D., Rifai N. (2007). C-Reactive Protein: A Nontraditional Serum Marker of Cardiovascular Risk. Cardiovasc. Pathol. Off. J. Soc. Cardiovasc. Pathol..

[5] Ong S.-B., Hernández-Reséndiz S., Crespo-Avilan G.E., Mukhametshina R.T., Kwek X.-Y., Cabrera-Fuentes H.A., Hausenloy D.J. (2018). Inflammation Following Acute Myocardial Infarction: Multiple Players, Dynamic Roles, and Novel Therapeutic Opportunities. Pharmacol. Ther..

[6] Anzai A., Ko S., Fukuda K. (2022). Immune and Inflammatory Networks in Myocardial Infarction: Current Research and Its Potential Implications for the Clinic. Int. J. Mol. Sci..

[7] Kologrivova I., Shtatolkina M., Suslova T., Ryabov V. (2021). Cells of the Immune System in Cardiac Remodeling: Main Players in Resolution of Inflammation and Repair After Myocardial Infarction. Front. Immunol..

[8] Andreadou I., Cabrera-Fuentes H.A., Devaux Y., Frangogiannis N.G., Frantz S., Guzik T., Liehn E.A., Gomes C.P.C., Schulz R., Hausenloy D.J. (2019). Immune Cells as Targets for Cardioprotection: New Players and Novel Therapeutic Opportunities. Cardiovasc. Res..

[9] Ma Y. (2021). Role of Neutrophils in Cardiac Injury and Repair Following Myocardial Infarction. Cells.

[10] Carbone F., Bonaventura A., Montecucco F. (2020). Neutrophil-Related Oxidants Drive Heart and Brain Remodeling After Ischemia/Reperfusion Injury. Front. Physiol..

[11] Heger L.A., Schommer N., Van Bruggen S., Sheehy C.E., Chan W., Wagner D.D. (2024). Neutrophil NLRP3 Promotes Cardiac Injury Following Acute Myocardial Infarction through IL-1β Production, VWF Release and NET Deposition in the Myocardium. Sci. Rep..

[12] O’Rourke S.A., Dunne A., Monaghan M.G. (2019). The Role of Macrophages in the Infarcted Myocardium: Orchestrators of ECM Remodeling. Front. Cardiovasc. Med..

[13] Murray P.J., Allen J.E., Biswas S.K., Fisher E.A., Gilroy D.W., Goerdt S., Gordon S., Hamilton J.A., Ivashkiv L.B., Lawrence T. (2014). Macrophage Activation and Polarization: Nomenclature and Experimental Guidelines. Immunity.

[14] Hausenloy D.J., Chilian W., Crea F., Davidson S.M., Ferdinandy P., Garcia-Dorado D., van Royen N., Schulz R., Heusch G. (2019). The Coronary Circulation in Acute Myocardial Ischaemia/Reperfusion Injury: A Target for Cardioprotection. Cardiovasc. Res..

[15] Yin W., Chen Y., Wang W., Guo M., Tong L., Zhang M., Wang Z., Yuan H. (2024). Macrophage-Mediated Heart Repair and Remodeling: A Promising Therapeutic Target for Post-Myocardial Infarction Heart Failure. J. Cell. Physiol..

[16] Martínez-Shio E.B., Marín-Jáuregui L.S., Rodríguez-Ortega A.C., Doníz-Padilla L.M., González-Amaro R., Escobedo-Uribe C.D., Monsiváis-Urenda A.E. (2024). Regulatory T-Cell Frequency and Function in Acute Myocardial Infarction Patients and Its Correlation with Ventricular Dysfunction. Clin. Exp. Immunol..

[17] Li Q., Mei A., Qian H., Min X., Yang H., Zhong J., Li C., Xu H., Chen J. (2023). The Role of Myeloid-Derived Immunosuppressive Cells in Cardiovascular Disease. Int. Immunopharmacol..

[18] Zhang M., Shi X., Zhao J., Guo W., Zhou J. (2023). Recruitment of Myeloid-derived Suppressor Cells and Regulatory T-cells Is Associated with the Occurrence of Acute Myocardial Infarction. Biomed. Rep..

[19] Chalise U., Becirovic-Agic M., Lindsey M.L. (2023). The Cardiac Wound Healing Response to Myocardial Infarction. WIREs Mech. Dis..

[20] Alter C., Henseler A.-S., Owenier C., Hesse J., Ding Z., Lautwein T., Bahr J., Hayat S., Kramann R., Kostenis E. (2023). IL-6 in the Infarcted Heart Is Preferentially Formed by Fibroblasts and Modulated by Purinergic Signaling. J. Clin. Investig..

[21] Wang J., Wang M., Lu X., Zhang Y., Zeng S., Pan X., Zhou Y., Wang H., Chen N., Cai F. (2022). IL-6 Inhibitors Effectively Reverse Post-Infarction Cardiac Injury and Ischemic Myocardial Remodeling via the TGF-Β1/Smad3 Signaling Pathway. Exp. Ther. Med..

[22] Deshmane S.L., Kremlev S., Amini S., Sawaya B.E. (2009). Monocyte Chemoattractant Protein-1 (MCP-1): An Overview. J. Interferon Cytokine Res..

[23] Frangogiannis N.G., Mendoza L.H., Lindsey M.L., Ballantyne C.M., Michael L.H., Smith C.W., Entman M.L. (2000). IL-10 Is Induced in the Reperfused Myocardium and May Modulate the Reaction to Injury. J. Immunol..

[24] Frangogiannis N.G. (2017). The Role of Transforming Growth Factor (TGF)-β in the Infarcted Myocardium. J. Thorac. Dis..

[25] Jung M., Ma Y., Iyer R.P., DeLeon-Pennell K.Y., Yabluchanskiy A., Garrett M.R., Lindsey M.L. (2017). IL-10 Improves Cardiac Remodeling after Myocardial Infarction by Stimulating M2 Macrophage Polarization and Fibroblast Activation. Basic Res. Cardiol..

[26] Neurath M.F., Finotto S. (2011). IL-6 Signaling in Autoimmunity, Chronic Inflammation and Inflammation-Associated Cancer. Cytokine Growth Factor. Rev..

[27] Tian M., Yuan Y.-C., Li J.-Y., Gionfriddo M.R., Huang R.-C. (2015). Tumor Necrosis Factor-α and Its Role as a Mediator in Myocardial Infarction: A Brief Review. Chronic Dis. Transl. Med..

[28] Krishnamurthy P., Rajasingh J., Lambers E., Qin G., Losordo D.W., Kishore R. (2009). IL-10 Inhibits Inflammation and Attenuates Left Ventricular Remodeling after Myocardial Infarction via Activation of STAT3 and Suppression of HuR. Circ. Res..

[29] Prabhu S.D., Frangogiannis N.G. (2016). The Biological Basis for Cardiac Repair After Myocardial Infarction: From Inflammation to Fibrosis. Circ. Res..

[30] Chen L., Deng H., Cui H., Fang J., Zuo Z., Deng J., Li Y., Wang X., Zhao L. (2017). Inflammatory Responses and Inflammation-Associated Diseases in Organs. Oncotarget.

[31] Baruah M., Ansar W., Ghosh S. (2020). C-Reactive Protein (CRP) and Markers of Oxidative Stress in Acute Myocardial Infarction. Clinical Significance of C-reactive Protein.

[32] Berg K., Jynge P., Bjerve K., Skarra S., Basu S., Wiseth R. (2005). Oxidative Stress and Inflammatory Response during and Following Coronary Interventions for Acute Myocardial Infarction. Free Radic. Res..

[33] Zheng Y., Xu L., Dong N., Li F. (2022). NLRP3 Inflammasome: The Rising Star in Cardiovascular Diseases. Front. Cardiovasc. Med..

[34] Mauro A.G., Bonaventura A., Mezzaroma E., Quader M., Toldo S. (2019). NLRP3 Inflammasome in Acute Myocardial Infarction. J. Cardiovasc. Pharmacol..

[35] Thackeray J.T. (2021). Molecular Imaging Using Cardiac PET/CT: Opportunities to Harmonize Diagnosis and Therapy. Curr. Cardiol. Rep..

[36] Saxena A., Russo I., Frangogiannis N.G. (2016). Inflammation as a Therapeutic Target in Myocardial Infarction: Learning from Past Failures to Meet Future Challenges. Transl. Res..

[37] Shimamoto A., Chong A.J., Yada M., Shomura S., Takayama H., Fleisig A.J., Agnew M.L., Hampton C.R., Rothnie C.L., Spring D.J. (2006). Inhibition of Toll-like Receptor 4 with Eritoran Attenuates Myocardial Ischemia-Reperfusion Injury. Circulation.

[38] Yang Y., Lv J., Jiang S., Ma Z., Wang D., Hu W., Deng C., Fan C., Di S., Sun Y. (2016). The Emerging Role of Toll-like Receptor 4 in Myocardial Inflammation. Cell Death Dis..

[39] Zhao L., Yang X.R., Han X. (2019). MicroRNA-146b Induces the PI3K/Akt/NF-κB Signaling Pathway to Reduce Vascular Inflammation and Apoptosis in Myocardial Infarction by Targeting PTEN. Exp. Ther. Med..

[40] Mitsis A., Kadoglou N.P.E., Lambadiari V., Alexiou S., Theodoropoulos K.C., Avraamides P., Kassimis G. (2022). Prognostic Role of Inflammatory Cytokines and Novel Adipokines in Acute Myocardial Infarction: An Updated and Comprehensive Review. Cytokine.

[41] Liu Y., Jia S., Yao Y., Tang X.-F., Xu N., Jiang L., Gao Z., Chen J., Yang Y.-J., Gao R.-L. (2020). Impact of High-Sensitivity C-Reactive Protein on Coronary Artery Disease Severity and Outcomes in Patients Undergoing Percutaneous Coronary Intervention. J. Cardiol..

[42] Carrero J.J., Andersson Franko M., Obergfell A., Gabrielsen A., Jernberg T. (2019). hsCRP Level and the Risk of Death or Recurrent Cardiovascular Events in Patients with Myocardial Infarction: A Healthcare-Based Study. J. Am. Heart Assoc..

[43] Ridker P.M., Buring J.E., Rifai N., Cook N.R. (2007). Development and Validation of Improved Algorithms for the Assessment of Global Cardiovascular Risk in Women: The Reynolds Risk Score. JAMA.

[44] Ridker P.M., Cook N. (2004). Clinical Usefulness of Very High and Very Low Levels of C-Reactive Protein across the Full Range of Framingham Risk Scores. Circulation.

[45] Ridker P.M., JUPITER Study Group (2003). Rosuvastatin in the Primary Prevention of Cardiovascular Disease among Patients with Low Levels of Low-Density Lipoprotein Cholesterol and Elevated High-Sensitivity C-Reactive Protein: Rationale and Design of the JUPITER Trial. Circulation.

[46] Pasceri V., Chang J.S., Willerson J.T., Yeh E.T. (2001). Modulation of C-Reactive Protein-Mediated Monocyte Chemoattractant Protein-1 Induction in Human Endothelial Cells by Anti-Atherosclerosis Drugs. Circulation.

[47] Limijadi E.K.S., Setyadi A., Utami S.B., Puruhito B., Sofia S.N. (2020). The Correlation between Neutrophil Lymphocyte Ratio, C-Reactive Protein, and Serum Amyloid a with the Degree of Stenosis in Acute Coronary Syndrome. Open Access Maced. J. Med. Sci..

[48] Sánchez P.L., Rodríguez M.V., Villacort E., Albarrán C., Cruz I., Moreiras J.M., Martín F., Pabón P., Fernández-Avilés F., Martín-Luengo C. (2006). Kinetics of C-reactive protein release in different forms of acute coronary syndrome. Rev. Esp. Cardiol..

[49] de Ferranti S., Rifai N. (2002). C-Reactive Protein and Cardiovascular Disease: A Review of Risk Prediction and Interventions. Clin. Chim. Acta.

[50] Maier W., Altwegg L.A., Corti R., Gay S., Hersberger M., Maly F.E., Sütsch G., Roffi M., Neidhart M., Eberli F.R. (2005). Inflammatory Markers at the Site of Ruptured Plaque in Acute Myocardial Infarction: Locally Increased Interleukin-6 and Serum Amyloid A but Decreased C-Reactive Protein. Circulation.

[51] Mueller C., Buettner H.J., Hodgson J.M., Marsch S., Perruchoud A.P., Roskamm H., Neumann F.-J. (2002). Inflammation and Long-Term Mortality after Non-ST Elevation Acute Coronary Syndrome Treated with a Very Early Invasive Strategy in 1042 Consecutive Patients. Circulation.

[52] Biasucci L.M., Liuzzo G., Grillo R.L., Caligiuri G., Rebuzzi A.G., Buffon A., Summaria F., Ginnetti F., Fadda G., Maseri A. (1999). Elevated Levels of C-Reactive Protein at Discharge in Patients with Unstable Angina Predict Recurrent Instability. Circulation.

[53] Karadeniz M., Duran M., Akyel A., Yarlıoğlueş M., Öcek A.H., Çelik İ.E., Kılıç A., Yalcin A.A., Ergün G., Murat S.N. (2015). High Sensitive CRP Level Is Associated with Intermediate and High Syntax Score in Patients with Acute Coronary Syndrome. Int. Heart J..

[54] Polyakova E.A., Mikhaylov E.N. (2020). The Prognostic Role of High-Sensitivity C-Reactive Protein in Patients with Acute Myocardial Infarction. J. Geriatr. Cardiol..

[55] SaranyaDevi K.S., Rekha B.S., Thiyagarajan J.V., Dhivya R., Mihiran S., Santhosh S. (2022). Comparative Evaluation of Salivary and Serum High-Sensitive C-Reactive Protein in Acute Myocardial Infarction. J. Pharm. Bioallied Sci..

[56] Domenico T., Rita A., Giacomo S., Diego A., Thelma P., Mariana G., Giampaolo N., Francesco N., Maria G., Francesco F. (2023). Salivary Biomarkers for Diagnosis of Acute Myocardial Infarction: A Systematic Review. Int. J. Cardiol..

[57] Miller C.S., Foley J.D., Floriano P.N., Christodoulides N., Ebersole J.L., Campbell C.L., Bailey A.L., Rose B.G., Kinane D.F., Novak M.J. (2014). Utility of Salivary Biomarkers for Demonstrating Acute Myocardial Infarction. J. Dent. Res..

[58] Shishehbor M.H., Bhatt D.L., Topol E.J. (2003). Using C-Reactive Protein to Assess Cardiovascular Disease Risk. Clevel. Clin. J. Med..

[59] Quispe R., Michos E.D., Martin S.S., Puri R., Toth P.P., Al Suwaidi J., Banach M., Virani S.S., Blumenthal R.S., Jones S.R. (2020). High-Sensitivity C-Reactive Protein Discordance with Atherogenic Lipid Measures and Incidence of Atherosclerotic Cardiovascular Disease in Primary Prevention: The ARIC Study. J. Am. Heart Assoc..

[60] Aday A.W., Ridker P.M. (2019). Targeting Residual Inflammatory Risk: A Shifting Paradigm for Atherosclerotic Disease. Front. Cardiovasc. Med..

[61] Pereira J., Ribeiro A., Ferreira-Coimbra J., Barroso I., Guimarães J.-T., Bettencourt P., Lourenço P. (2018). Is There a C-Reactive Protein Value beyond Which One Should Consider Infection as the Cause of Acute Heart Failure?. BMC Cardiovasc. Disord..

[62] Ørn S., Manhenke C., Ueland T., Damås J.K., Mollnes T.E., Edvardsen T., Aukrust P., Dickstein K. (2009). C-Reactive Protein, Infarct Size, Microvascular Obstruction, and Left-Ventricular Remodelling Following Acute Myocardial Infarction. Eur. Heart J..

[63] Reindl M., Reinstadler S.J., Feistritzer H.-J., Klug G., Tiller C., Mair J., Mayr A., Jaschke W., Metzler B. (2017). Relation of Inflammatory Markers with Myocardial and Microvascular Injury in Patients with Reperfused ST-Elevation Myocardial Infarction. Eur. Heart J. Acute Cardiovasc. Care.

[64] Bursi F., Weston S.A., Killian J.M., Gabriel S.E., Jacobsen S.J., Roger V.L. (2007). C-Reactive Protein and Heart Failure after Myocardial Infarction in the Community. Am. J. Med..

[65] Griselli M., Herbert J., Hutchinson W.L., Taylor K.M., Sohail M., Krausz T., Pepys M.B. (1999). C-Reactive Protein and Complement Are Important Mediators of Tissue Damage in Acute Myocardial Infarction. J. Exp. Med..

[66] Ribeiro D.R.P., Ramos A.M., Vieira P.L., Menti E., Bordin O.L., de Souza P.A.L., de Quadros A.S., Portal V.L. (2014). High-Sensitivity C-Reactive Protein as a Predictor of Cardiovascular Events after ST-Elevation Myocardial Infarction. Arq. Bras. Cardiol..

[67] Akkus M.N., Polat G., Yurtdas M., Akcay B., Ercetin N., Cicek D., Doven O., Sucu N. (2009). Admission Levels of C-Reactive Protein and Plasminogen Activator Inhibitor-1 in Patients with Acute Myocardial Infarction with and without Cardiogenic Shock or Heart Failure on Admission. Int. Heart J..

[68] Zhu Y., Yu Z., Xu R., Wang B., Lou Y., Zhang N., Chen Z. (2023). Associations of Serum High-Sensitivity C-Reactive Protein and Prealbumin with Coronary Vessels Stenosis Determined by Coronary Angiography and Heart Failure in Patients with Myocardial Infarction. J. Med. Biochem..

[69] Hodzic E., Drakovac A., Begic E. (2018). Troponin and CRP as Indicators of Possible Ventricular Arrhythmias in Myocardial Infarction of the Anterior and Inferior Walls of the Heart. Mater. Sociomed..

[70] Kobayashi Y., Tanno K., Ueno A., Fukamizu S., Murata H., Watanabe N., Sasaki T., Yamamoto T., Takayama M., Nagao K. (2018). In-Hospital Electrical Storm in Acute Myocardial Infarction—Clinical Background and Mechanism of the Electrical Instability. Circ. J..

[71] Świątkiewicz I., Magielski P., Kubica J. (2021). C-Reactive Protein as a Risk Marker for Post-Infarct Heart Failure over a Multi-Year Period. Int. J. Mol. Sci..

[72] Sharif D., Hammoud M., Sharif-Rasslan A., Abinader E., Odeh M. (2015). Very Early C-Reactive Protein Levels after Acute Myocardial Infarction Predict Early Outcome and Late Prognosis. Int. J. Clin. Med..

[73] Morishima I., Sone T., Tsuboi H., Kondo J., Mukawa H., Kamiya H., Hieda N., Okumura K. (2002). Plasma C-reactive Protein Predicts Left Ventricular Remodeling and Function after a First Acute Anterior Wall Myocardial Infarction Treated with Coronary Angioplasty: Comparison with Brain Natriuretic Peptide. Clin. Cardiol..

[74] Al Aseri Z.A., Habib S.S., Marzouk A. (2019). Predictive Value of High Sensitivity C-Reactive Protein on Progression to Heart Failure Occurring after the First Myocardial Infarction. Vasc. Health Risk Manag..

[75] Arruda-Olson A.M., Enriquez-Sarano M., Bursi F., Weston S.A., Jaffe A.S., Killian J.M., Roger V.L. (2010). Left Ventricular Function and C-Reactive Protein Levels in Acute Myocardial Infarction. Am. J. Cardiol..

[76] Engelen S.E., Robinson A.J.B., Zurke Y.-X., Monaco C. (2022). Therapeutic Strategies Targeting Inflammation and Immunity in Atherosclerosis: How to Proceed?. Nat. Rev. Cardiol..

[77] Kayser S., Brunner P., Althaus K., Dorst J., Sheriff A. (2020). Selective Apheresis of C-Reactive Protein for Treatment of Indications with Elevated CRP Concentrations. J. Clin. Med..

[78] Slagman A.C., Bock C., Abdel-Aty H., Vogt B., Gebauer F., Janelt G., Wohlgemuth F., Morgenstern R., Yapici G., Puppe A. (2011). Specific Removal of C-Reactive Protein by Apheresis in a Porcine Cardiac Infarction Model. Blood Purif..

[79] Sheriff A., Schindler R., Vogt B., Abdel-Aty H., Unger J.K., Bock C., Gebauer F., Slagman A., Jerichow T., Mans D. (2015). Selective Apheresis of C-Reactive Protein: A New Therapeutic Option in Myocardial Infarction?. J. Clin. Apher..

[80] Mattecka S., Brunner P., Hähnel B., Kunze R., Vogt B., Sheriff A. (2019). PentraSorb C-Reactive Protein: Characterization of the Selective C-Reactive Protein Adsorber Resin. Ther. Apher. Dial..

[81] Torzewski J., Brunner P., Ries W., Garlichs C.D., Kayser S., Heigl F., Sheriff A. (2022). Targeting C-Reactive Protein by Selective Apheresis in Humans: Pros and Cons. J. Clin. Med..

[82] Ries W., Torzewski J., Heigl F., Pfluecke C., Kelle S., Darius H., Ince H., Mitzner S., Nordbeck P., Butter C. (2021). C-Reactive Protein Apheresis as Anti-Inflammatory Therapy in Acute Myocardial Infarction: Results of the CAMI-1 Study. Front. Cardiovasc. Med..

[83] Banach M., Penson P.E. (2021). Colchicine and Cardiovascular Outcomes: A Critical Appraisal of Recent Studies. Curr. Atheroscler. Rep..

[84] Tardif J.-C., Kouz S., Waters D.D., Bertrand O.F., Diaz R., Maggioni A.P., Pinto F.J., Ibrahim R., Gamra H., Kiwan G.S. (2019). Efficacy and Safety of Low-Dose Colchicine after Myocardial Infarction. N. Engl. J. Med..

[85] Nidorf S.M., Fiolet A.T.L., Mosterd A., Eikelboom J.W., Schut A., Opstal T.S.J., The S.H.K., Xu X.-F., Ireland M.A., Lenderink T. (2020). Colchicine in Patients with Chronic Coronary Disease. N. Engl. J. Med..

[86] Bouabdallaoui N., Blondeau L., Tardif J.-C. (2021). Lessons from COLCOT and LoDoCo2: Colchicine for Secondary Prevention in Coronary Artery Disease. Eur. Heart J..

[87] Ridker P.M., Everett B.M., Thuren T., MacFadyen J.G., Chang W.H., Ballantyne C., Fonseca F., Nicolau J., Koenig W., Anker S.D. (2017). Antiinflammatory Therapy with Canakinumab for Atherosclerotic Disease. N. Engl. J. Med..

[88] Shah S.R., Abbasi Z., Fatima M., Ochani R.K., Shahnawaz W., Asim Khan M., Shah S.A. (2018). Canakinumab and Cardiovascular Outcomes: Results of the CANTOS Trial. J. Community Hosp. Intern. Med. Perspect..

[89] Ko S.-F., Yip H.-K., Leu S., Lee C.-C., Sheu J.-J., Lee C.-C., Ng S.-H., Huang C.-C., Chen M.-C., Sun C.-K. (2014). Therapeutic Potential of Tacrolimus on Acute Myocardial Infarction in Minipigs: Analysis with Serial Cardiac Magnetic Resonance and Changes at Histological and Protein Levels. Biomed. Res. Int..

[90] Elezaby A., Dexheimer R., Sallam K. (2022). Cardiovascular Effects of Immunosuppression Agents. Front. Cardiovasc. Med..

[91] Sheu J.-J., Sung P.-H., Leu S., Chai H.-T., Zhen Y.-Y., Chen Y.-C., Chua S., Chen Y.-L., Tsai T.-H., Lee F.-Y. (2013). Innate Immune Response after Acute Myocardial Infarction and Pharmacomodulatory Action of Tacrolimus in Reducing Infarct Size and Preserving Myocardial Integrity. J. Biomed. Sci..

[92] Guo Q., Jin Y., Chen X., Ye X., Shen X., Lin M., Zeng C., Zhou T., Zhang J. (2024). NF-κB in Biology and Targeted Therapy: New Insights and Translational Implications. Signal Transduct. Target. Ther..

[93] Alisky J.M. (2006). Dexamethasone Could Improve Myocardial Infarction Outcomes and Provide New Therapeutic Options for Non-Interventional Patients. Med. Hypotheses.

[94] Cheng X., Zhang R., Wei S., Huang J., Zhai K., Li Y., Gao B. (2022). Dexamethasone Alleviates Myocardial Injury in a Rat Model of Acute Myocardial Infarction Supported by Venoarterial Extracorporeal Membrane Oxygenation. Front. Public Health.

[95] Giugliano G.R., Giugliano R.P., Gibson C.M., Kuntz R.E. (2003). Meta-Analysis of Corticosteroid Treatment in Acute Myocardial Infarction. Am. J. Cardiol..

[96] Tan F., Li X., Wang Z., Li J., Shahzad K., Zheng J. (2024). Clinical Applications of Stem Cell-Derived Exosomes. Signal Transduct. Target. Ther..

[97] Singla D.K., Johnson T.A., Tavakoli Dargani Z. (2019). Exosome Treatment Enhances Anti-Inflammatory M2 Macrophages and Reduces Inflammation-Induced Pyroptosis in Doxorubicin-Induced Cardiomyopathy. Cells.

[98] Naji A., Eitoku M., Favier B., Deschaseaux F., Rouas-Freiss N., Suganuma N. (2019). Biological Functions of Mesenchymal Stem Cells and Clinical Implications. Cell. Mol. Life Sci..

[99] Katsur M., He Z., Vinokur V., Corteling R., Yellon D.M., Davidson S.M. (2021). Exosomes from Neuronal Stem Cells May Protect the Heart from Ischaemia/Reperfusion Injury via JAK1/2 and Gp130. J. Cell. Mol. Med..

[100] Peng Y., Zhao J.-L., Peng Z.-Y., Xu W.-F., Yu G.-L. (2020). Exosomal miR-25-3p from Mesenchymal Stem Cells Alleviates Myocardial Infarction by Targeting pro-Apoptotic Proteins and EZH2. Cell Death Dis..

[101] Ghibu S., Richard C., Vergely C., Zeller M., Cottin Y., Rochette L. (2009). Antioxidant Properties of an Endogenous Thiol: Alpha-Lipoic Acid, Useful in the Prevention of Cardiovascular Diseases. J. Cardiovasc. Pharmacol..

[102] Salehi B., Berkay Yılmaz Y., Antika G., Boyunegmez Tumer T., Fawzi Mahomoodally M., Lobine D., Akram M., Riaz M., Capanoglu E., Sharopov F. (2019). Insights on the Use of α-Lipoic Acid for Therapeutic Purposes. Biomolecules.

[103] Feng X., Sureda A., Jafari S., Memariani Z., Tewari D., Annunziata G., Barrea L., Hassan S.T.S., Šmejkal K., Malaník M. (2019). Berberine in Cardiovascular and Metabolic Diseases: From Mechanisms to Therapeutics. Theranostics.

[104] Ruan J., Shi Z., Cao X., Dang Z., Zhang Q., Zhang W., Wu L., Zhang Y., Wang T. (2024). Research Progress on Anti-Inflammatory Effects and Related Mechanisms of Astragalin. Int. J. Mol. Sci..

[105] Dinarello C.A. (2004). Unraveling the NALP-3/IL-1beta Inflammasome: A Big Lesson from a Small Mutation. Immunity.

[106] Yao J., Sterling K., Wang Z., Zhang Y., Song W. (2024). The Role of Inflammasomes in Human Diseases and Their Potential as Therapeutic Targets. Signal Transduct. Target. Ther..

[107] Shen S., Wang Z., Sun H., Ma L. (2022). Role of NLRP3 Inflammasome in Myocardial Ischemia-Reperfusion Injury and Ventricular Remodeling. Med. Sci. Monit..

[108] Toldo S., Mezzaroma E., Buckley L.F., Potere N., Di Nisio M., Biondi-Zoccai G., Van Tassell B.W., Abbate A. (2022). Targeting the NLRP3 Inflammasome in Cardiovascular Diseases. Pharmacol. Ther..

[109] Koenig W., Khuseyinova N. (2009). Lipoprotein-Associated and Secretory Phospholipase A2 in Cardiovascular Disease: The Epidemiological Evidence. Cardiovasc. Drugs Ther..

[110] Saxena J., Das S., Kumar A., Sharma A., Sharma L., Kaushik S., Kumar Srivastava V., Jamal Siddiqui A., Jyoti A. (2024). Biomarkers in Sepsis. Clin. Chim. Acta.

[111] Szalai A.J. (2004). C-Reactive Protein (CRP) and Autoimmune Disease: Facts and Conjectures. Clin. Dev. Immunol..

[112] Li J., Chen J., Lan H., Tang Y. (2022). Role of C-Reactive Protein in Kidney Diseases. Kidney Dis..

[113] Hart P.C., Rajab I.M., Alebraheem M., Potempa L.A. (2020). C-Reactive Protein and Cancer—Diagnostic and Therapeutic Insights. Front. Immunol..

[114] Visser M., Bouter L.M., McQuillan G.M., Wener M.H., Harris T.B. (1999). Elevated C-Reactive Protein Levels in Overweight and Obese Adults. JAMA.

[115] Neumaier M., Metak G., Scherer M.A. (2006). C-Reactive Protein as a Parameter of Surgical Trauma: CRP Response after Different Types of Surgery in 349 Hip Fractures. Acta Orthop..

[116] Brzezinski R.Y., Melloul A., Berliner S., Goldiner I., Stark M., Rogowski O., Banai S., Shenhar-Tsarfaty S., Shacham Y. (2022). Early Detection of Inflammation-Prone STEMI Patients Using the CRP Troponin Test (CTT). J. Clin. Med..

[117] Osman R., L’Allier P.L., Elgharib N., Tardif J.-C. (2006). Critical Appraisal of C-Reactive Protein Throughout the Spectrum of Cardiovascular Disease. Vasc. Health Risk Manag..

[118] Matter M.A., Paneni F., Libby P., Frantz S., Stähli B.E., Templin C., Mengozzi A., Wang Y.-J., Kündig T.M., Räber L. (2024). Inflammation in Acute Myocardial Infarction: The Good, the Bad and the Ugly. Eur. Heart J..

[119] Mitsis A., Kyriakou M., Sokratous S., Karmioti G., Drakomathioulakis M., Myrianthefs M., Ziakas A., Tzikas S., Kassimis G. (2024). Exploring the Landscape of Anti-Inflammatory Trials: A Comprehensive Review of Strategies for Targeting Inflammation in Acute Myocardial Infraction. Biomedicines.

[120] Mitchell A.M., Garvey J.L., Kline J.A. (2006). Multimarker Panel to Rule out Acute Coronary Syndromes in Low-Risk Patients. Acad. Emerg. Med..

[121] Ritschel V.N., Seljeflot I., Arnesen H., Halvorsen S., Weiss T., Eritsland J., Andersen G.Ø. (2014). IL-6 Signalling in Patients with Acute ST-Elevation Myocardial Infarction. Results Immunol..

[122] Rafiqi K., Hoeks C.B., Løfgren B., Mortensen M.B., Bruun J.M. (2023). Diagnostic Impact of Hs-CRP and IL-6 for Acute Coronary Syndrome in Patients Admitted to the ED with Chest Pain: Added Value to the HEART Score?. Open Access Emerg. Med..

[123] Kelly D., Khan S.Q., Dhillon O., Quinn P., Struck J., Squire I.B., Davies J.E., Ng L.L. (2010). Procalcitonin as a Prognostic Marker in Patients with Acute Myocardial Infarction. Biomarkers.

[124] Reindl M., Tiller C., Holzknecht M., Lechner I., Henninger B., Mayr A., Brenner C., Klug G., Bauer A., Metzler B. (2020). Association of Myocardial Injury with Serum Procalcitonin Levels in Patients With ST-Elevation Myocardial Infarction. JAMA Netw. Open.

[125] Yu L., Sun J., Liu X. (2023). Serum C Reactive Protein and Procalcitonin Are Valuable Predictors of Coronary Heart Disease and Poor Prognosis in the Elderly. Am. J. Transl. Res..

[126] Churov A., Summerhill V., Grechko A., Orekhova V., Orekhov A. (2019). MicroRNAs as Potential Biomarkers in Atherosclerosis. Int. J. Mol. Sci..

[127] Kim E.N., Kim C.J., Kim S.R., Song J.-A., Choe H., Kim K.-B., Choi J.-S., Oh S.J. (2019). High Serum CRP Influences Myocardial miRNA Profiles in Ischemia-Reperfusion Injury of Rat Heart. PLoS ONE.

[128] Kristono G., Holley A., Harding S., Larsen P. (2021). Using C-Reactive Protein and Cytokines to Identify Inflammatory States in Acute Myocardial Infarction. Heart Lung Circ..

[129] Mitsis A., Myrianthefs M., Sokratous S., Karmioti G., Kyriakou M., Drakomathioulakis M., Tzikas S., Kadoglou N.P.E., Karagiannidis E., Nasoufidou A. (2024). Emerging Therapeutic Targets for Acute Coronary Syndromes: Novel Advancements and Future Directions. Biomedicines.

[130] Amezcua-Castillo E., González-Pacheco H., Sáenz-San Martín A., Méndez-Ocampo P., Gutierrez-Moctezuma I., Massó F., Sierra-Lara D., Springall R., Rodríguez E., Arias-Mendoza A. (2023). C-Reactive Protein: The Quintessential Marker of Systemic Inflammation in Coronary Artery Disease—Advancing toward Precision Medicine. Biomedicines.

[131] Ridker P.M., Rifai N., Clearfield M., Downs J.R., Weis S.E., Miles J.S., Gotto A.M., Air Force/Texas Coronary Atherosclerosis Prevention Study Investigators (2001). Measurement of C-Reactive Protein for the Targeting of Statin Therapy in the Primary Prevention of Acute Coronary Events. N. Engl. J. Med..

[132] Schuetz P., Aujesky D., Müller C., Müller B. (2015). Biomarker-Guided Personalised Emergency Medicine for All—Hope for Another Hype?. Swiss Med. Wkly..

[133] Burgos L.M., Baro Vila R.C., Botto F., Diez M. (2022). SCAI Cardiogenic Shock Classification for Predicting In-Hospital and Long-Term Mortality in Acute Heart Failure. J. Soc. Cardiovasc. Angiogr. Interv..

[134] Aladağ N., Asoğlu R., Ozdemir M., Asoğlu E., Derin A.R., Demir C., Demir H. (2021). Oxidants and Antioxidants in Myocardial Infarction (MI): Investigation of Ischemia Modified Albumin, Malondialdehyde, Superoxide Dismutase and Catalase in Individuals Diagnosed with ST Elevated Myocardial Infarction (STEMI) and Non-STEMI (NSTEMI). J. Med. Biochem..

[135] Silvis M.J.M., Demkes E.J., Fiolet A.T.L., Dekker M., Bosch L., van Hout G.P.J., Timmers L., de Kleijn D.P.V. (2021). Immunomodulation of the NLRP3 Inflammasome in Atherosclerosis, Coronary Artery Disease, and Acute Myocardial Infarction. J. Cardiovasc. Transl. Res..

[136] Toldo S., Mauro A.G., Cutter Z., Van Tassell B.W., Mezzaroma E., Del Buono M.G., Prestamburgo A., Potere N., Abbate A. (2019). The NLRP3 Inflammasome Inhibitor, OLT1177 (Dapansutrile), Reduces Infarct Size and Preserves Contractile Function After Ischemia Reperfusion Injury in the Mouse. J. Cardiovasc. Pharmacol..

[137] Shi Y., Zhang J., Tan C., Xu W., Sun Q., Li J. (2015). Genetic Association Studies Reporting on Variants in the C-Reactive Protein Gene and Coronary Artery Disease. Medicine.

[138] Schulz S., Rehm S., Schlitt A., Lierath M., Lüdike H., Hofmann B., Bitter K., Reichert S. (2023). C-Reactive Protein Level and the Genetic Variant Rs1130864 in the CRP Gene as Prognostic Factors for 10-Year Cardiovascular Outcome. Cells.

[139] Nie S., Zhang S., Zhao Y., Li X., Xu H., Wang Y., Wang X., Zhu M. (2025). Machine Learning Applications in Acute Coronary Syndrome: Diagnosis, Outcomes and Management. Adv. Ther..

